# Ventilatory response to added dead space in infants exposed to second-hand smoke in pregnancy

**DOI:** 10.1007/s00431-023-04991-5

**Published:** 2023-05-11

**Authors:** Allan Jenkinson, Nadja Bednarczuk, Ourania Kaltsogianni, Emma E. Williams, Rebecca Lee, Ravindra Bhat, Theodore Dassios, Anthony D. Milner, Anne Greenough

**Affiliations:** 1 Department of Women and Children’s Health, School of Life Course Sciences, Faculty of Life Sciences and Medicine, King’s College London, King’s College Hospital NHS Foundation Trust, 4th Floor Golden Jubilee Wing, Denmark Hill, London, SE5 9RS UK; 2 Neonatal Intensive Care Centre, King’s College Hospital NHS Foundation Trust, London, UK

**Keywords:** Minute ventilation, Nicotine, Antenatal smoke exposure, Maternal smoking, Respiratory control

## Abstract

Maternal cigarette smoking in pregnancy can adversely affect infant respiratory control. In utero nicotine exposure has been shown to blunt the infant ventilatory response to hypercapnia, which could increase the risk of sudden infant death syndrome. The potential impact of maternal second-hand smoke exposure, however, has not yet been determined. The aim of this study was to assess ventilatory response to added dead-space (inducing hypercapnia) in infants with second-hand smoke exposure during pregnancy, in infants whose mothers smoked and in controls (non-smoke exposed). Infants breathed through a face mask and specialised “tube-breathing” circuit, incorporating a dead space of 4.4 ml/kg body weight. The maximum minute ventilation (MMV) during added dead space breathing was determined and the time taken to achieve 63% of the MMV calculated (the time constant (TC) of the response). Infants were studied on the postnatal ward prior to discharge home. Thirty infants (ten in each group) were studied with a median gestational age of 39 [range 37–41] weeks, birthweight of 3.1 [2.2–4.0] kg, and postnatal age of 33 (21–62) h. The infants whose mothers had second-hand smoke exposure (median TC 42 s, *p* = 0.001), and the infants of cigarette smoking mothers (median TC 37 s, *p* = 0.002) had longer time constants than the controls (median TC 29 s). There was no significant difference between the TC of the infants whose mothers had second-hand smoke exposure and those whose mothers smoked (*p* = 0.112).

*    Conclusion*: Second-hand smoke exposure during pregnancy was associated with a delayed newborn ventilatory response.

**Table:** 

**What is Known:** *• Maternal cigarette smoking in pregnancy can adversely affect infant respiratory control*. *• The potential impact of maternal second-hand smoke exposure, however, has not yet been determined*.	
**What is New:** *• We have assessed the ventilatory response to added dead-space (inducing hypercapnia) in newborns with second-hand smoke exposure during pregnancy, in infants whose mothers smoked, and in controls (non-smoke exposed)*. *• Maternal second-hand smoke exposure, as well as maternal smoking, during pregnancy was associated with a delayed newborn ventilatory response*.

**What is Known:**

*• Maternal cigarette smoking in pregnancy can adversely affect infant respiratory control*.

*• The potential impact of maternal second-hand smoke exposure, however, has not yet been determined*.

**What is New:**

*• We have assessed the ventilatory response to added dead-space (inducing hypercapnia) in newborns with second-hand smoke exposure during pregnancy, in infants whose mothers smoked, and in controls (non-smoke exposed)*.

*• Maternal second-hand smoke exposure, as well as maternal smoking, during pregnancy was associated with a delayed newborn ventilatory response*.

## Introduction

Maternal smoking during pregnancy is associated with abnormal lung function in childhood [1, 2]. Furthermore, infants whose mothers smoke during pregnancy are at increased risk of sudden infant death syndrome (SIDS) compared with infants of non-smoking mothers. The increase in risk has been reported to be twofold to fourfold, but as high as sixfold if associated with other risk factors [3–5]. Non-combustible nicotine (Swedish snuff) use in pregnancy has been shown to be associated with an increased risk of neonatal mortality and SIDS. Those findings support the hypothesis that nicotine contributes to an increased risk of SIDS [6]. A possible explanation is that such infants have a reduced ventilatory response to hypercarbia [7]. Indeed, term newborns of smoking mothers have a delayed response to an imposed dead space (tube breathing) and hypercarbia is the most important stimulus to ventilation during tube breathing [8, 9]. The aim of this study was to determine if maternal second-hand smoke exposure during pregnancy might also impact on infant’s ventilatory control in a similar manner to maternal smoking.

## Methods

Infants of mothers exposed to second-hand smoke during pregnancy, cigarette smoking mothers and non-smoking, and non-exposed mothers were recruited from the postnatal ward. Infants born at term receiving routine postnatal care, with no significant cardiac, respiratory, or congenital anomalies were included in this study. Mother’s smoking status, including their exposure to second-hand smoke, was ascertained by review of antenatal records and by discussion with the mothers. Mothers were classified as exposed to second-hand smoke if they did not smoke themselves, but household members smoked. Maternal cigarette smoking was quantified to the nearest five cigarettes smoked per day. Urinary cotinine concentrations were not assessed. Gestational and post-natal age, birthweight, and delivery method were recorded for each infant. Only one of the infants fulfilled the definition of small for gestational age (SGA); we therefore reported the birth weight *z*-scores calculated using the UK-World Health Organization (WHO) preterm reference chart [10] and the Microsoft Excel add-in LMS Growth (version 2.77; www.healthforchildren.co.uk). The study was approved by the London Brent Research Ethics Committee, and parent(s) gave informed, written consent for their infant to take part.

### Tube breathing

The ventilatory response to added dead space was determined using a specialised breathing circuit incorporating a facemask placed over the infant’s nose and mouth. Infants were awake, but quiet, throughout the recorded study period. A bias flow of air (2 L/min) was delivered to the facemask using 7.0 mm internal diameter tubing via a three-way tap. A second tube (7.0 mm internal diameter) connected the face mask to a pneumotachograph (PK Morgan, Rainham Kent, UK) via a Y connector. The length of the tubing (between face mask and Y connector) was adjusted so that its internal volume was 4.4 ml/kg body weight, twice the anatomical dead space. A third tube connected the third port on the three-way tap to the remaining port of the Y connector. The pneumotachograph was attached to a differential pressure transducer (MP45m Validyne Cooperation, Northridge, CA, USA), from which the signal was amplified (DC280; Validyne Cooperation) and displayed in real time on a computer running the Spectra software (Grove Medical, London, UK), with 100 Hz analog to digital sampling (DAQ 16XE-50; National Instruments, Austin, TX, USA). Tidal volume was obtained by digital integration of the flow signal using the Spectra software, and minute volume, inspiratory, expiratory, and total respiratory cycle times were determined. Respiratory rate was calculated breath by breath. Oxygen saturations at baseline and during tube breathing were monitored. All infants had saturations greater than 95%.

**Fig. 1:**
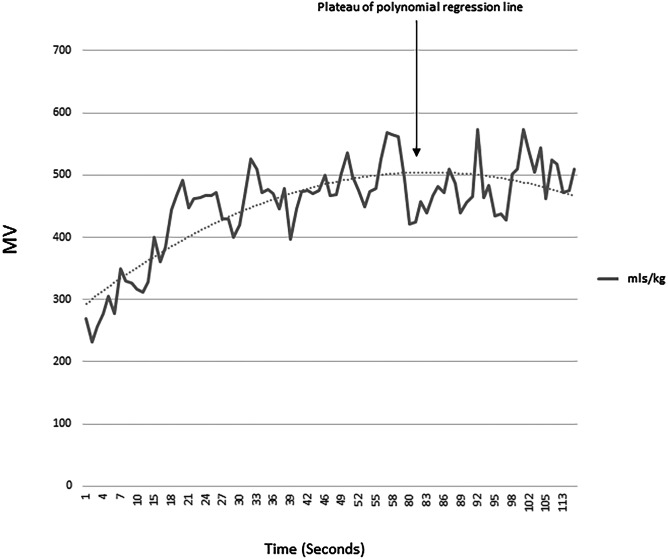
MV plotted against time; MMV is indicated by the arrow

Baseline recordings of respiratory flow were made initially with the three-way tap in the neutral position. In that position, the bias flow of 2 L/min of air to the face mask eliminated any dead space. When the tap was rotated so that the bias flow was fed via the third tube directly to the Y connector and pneumotachograph, the bias flow bypassed the facemask, which resulted in the dead space of the second tube being added to the infant’s respiratory system. The infant breathed through the additional dead space until the maximum minute ventilation was reached (Fig. 1). The response to added dead space was determined by calculating the time constant, the time taken to achieve 63% increase in minute ventilation. The baseline data was calculated from a minimum of ten breaths, and the MMV of tube breathing was calculated from 10 breaths.

### Statistical analysis

Data were assessed for normality using histogram and normality plot inspection. As data were found to be not normally distributed, the Mann–Whitney and Kruskal–Wallis tests were used to assess if differences between the groups were statistically significant. Post hoc tests were carried out using the Mann Whitney *U* test. Data analysis was performed using Stata 17.0 (StataCorp, College Station, TX, USA).

### Sample size

A previous study has shown that the mean time constant (time to achieve 63% increase in minute ventilation) in non-smoke exposed term infants was 26.2 s [8]. In smoking mothers, the time constant was significantly longer, at an average of 37.3 s [9]. Based upon these findings, recruitment of 10 infants per group allowed detection of a difference in the time constant of 11 s with at least 80% power at the 5% level.

## Results

A total of thirty infants (ten in each group) were studied with a median gestational age of 39 (range 37–41] weeks, birthweight of 3.1 [2.2–4.0] kg, and postnatal age of 31 (21–62) h. All mothers of the smoking group smoked at least five cigarettes/day (range 5–20 cigarettes/day). There were no significant differences in the birthweight (*p* = 0.53) or gestational age (*p* = 0.17) of the three groups (Table 1). The median (IQR) birth weight *z*-score was not significantly different between control [0.02 (− 0.77 to 0.35)], maternal smoking [0.00 (− 0.48 to 0.32)], and second-hand smoking [− 0.44 (− 1.11 to 0.21)] infants *p* = 0.705).

**Table 1:** Demographics by maternal smoke exposure. Data are expressed as median (IQR) or *N* (%)

	**Second-hand smoke exposure** ***N* = 10**	**Maternal smoking** ***N* = 10**	**Control** ***N* = 10**	***p*-value**
**Birthweight (kg)**	3.2 (3.0–3.4)	3.0 (2.7–3.5)	3.2 (3–3.4)	0.532
**Gestational age (weeks)**	39 (38–41)	38 (37–39)	39 (38–40)	0.177
**Male**	6 (60%)	7 (70%)	4 (40%)	0.387
**Postnatal age (h)**	25 (18–38)	26 (17–44)	31 (19–47)	0.840
**Vaginal delivery**	5 (50%)	3 (30%)	4 (40%)	0.581

**Fig. 2:**
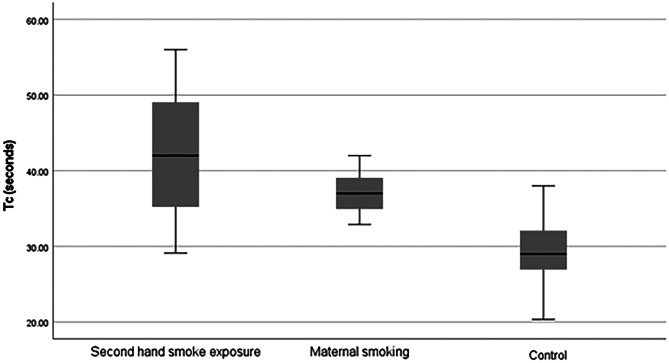
A box plot of the time constants of the response to added dead space by maternal smoking status

The second-hand smoke exposure group (*p* = 0.001) and the “smoking” group (*p* = 0.002) had longer time constants than the controls, but there was no significant difference between the time constants of the second-hand exposure and the smoking groups (*p* = 0.112) (Table 2; Fig. 2).

**Table 2:** Response to added space by maternal smoking status. Data are expressed as median (interquartile range)

	**Second-hand smoke exposure** ***N* = 10**	**Maternal smoking** ***N* = 10**	**Control** ***N* = 10**	***p*-value**
**Baseline RR (breaths/minute)**	46 (40–57)	59 (44–64)	52 (42–57)	0.205
**Baseline TV (ml/kg)**	5.6 (4.9–6.3)	5.2 (4.6–5.7)	5.3 (4.7–6.0)	0.602
**Baseline MV (ml/min/kg)**	274 (223–328)	284 (213–336)	264 (218–325)	0.931
**Added dead space RR (breaths/minute)**	61 (53–74)	70 (60–80)	62 (55–69)	0.348
**Added dead space TV (ml/kg)**	8.6 (8–9.3)	7.9 (7.5–8.5)	7.9 (6.9–9.1)	0.194
**MMV (ml/min/kg)**	493 (411–514)	483 (391–563)	402 (364–445)	0.124
**TC (s)**	42 (34–49)	37 (34–39.5)	29 (26–33)	< 0.001

## Discussion

We have demonstrated that infants born to mothers exposed to second-hand smoke exposure and those who smoked during pregnancy had a delayed ventilatory response to added dead space as evidenced by longer time constants (TC) than the controls. This may be the first time that antenatal, passive second-hand smoke exposure of mothers has been shown to result in a delayed ventilatory response in their infants. We examined infants while still on the postnatal ward and thus were examining the effect of antenatal exposure only and not postnatal smoke exposure.

Tobacco smoke exposure has been linked to an increased risk of sudden infant death syndrome (SIDS) [11]. The mechanisms underlying this link between prenatal tobacco smoke exposure and SIDS have not been clearly elucidated. Rodent studies have indicated that nicotine exposure during prenatal life may alter central and peripheral respiratory chemoreception [12, 13]. Our results showing a delayed respiratory response to added dead space (and therefore increased levels of CO_2_) suggest that infants of mothers with second-hand exposure to tobacco smoke, as well as infants of maternal smokers, may be maladapted to respond to suboptimal respiratory environments.

It has been demonstrated that children with maternal exposure to passive smoking during pregnancy and no other smoking exposure are more likely to develop wheeze up to the age of 2 years compared with unexposed children [14]. They are also more likely to suffer from otitis media and upper respiratory tract infections [15]. Maternal second-hand smoke exposure has consistently been shown to have a negative association with foetal growth, with lower weight, head circumference, and length all being reported [16, 17]. Placental studies have shown that mothers with passive smoking exposure had higher rates of placental hypoplasia and foetal vascular malperfusion [18]. A meta-analysis performed to assess the risks associated with passive smoking found that compared with smoking, exposure to passive smoking during pregnancy carries a higher risk of infant neural tube defects [19]. Thus, the adverse effect on infant ventilatory control of passive maternal smoking exposure we report is biologically plausible. There was no significant difference in the TCs of the infants in smoking and passive smoking groups. This may suggest that both types of exposure were similarly injurious, but the numbers in the two groups may have been too low to detect small differences between them.

This study has strengths and some limitations. We used a technique which has previously been used to assess infants of mothers who smoked during pregnancy [7]. Our time constant results for the infants of maternal smokers were very similar to the previous results [7]. We did not measure cotinine levels and relied on maternal report. This was, however, not only at the time of measurement but also recorded by the midwives during pregnancy. Previous work has shown that hypercarbia is the predominant factor that stimulates the infant’s ventilatory response during tube breathing [8]. Resistance is another influencing factor [8]. The faster the respiratory rates, the greater the effective additional dead space per unit time. Hence, the dead space tube was standardised for weight and there were no significant differences in respiratory rates between the groups. Additionally, sleep state has been suggested to influence the results [20]. Hence, all the infants were studied while awake, but quiet. A further limitation is that our sample size was too small to conduct multiple corrections in multivariate analyses, and thus, the observed results may be due to unnoticed factors such as social/health environment and illnesses connected to smoking close to the pregnant mother. There were, however, no significant differences in the infant demographics. We did not measure oxygen saturation or transcutaneous carbon dioxide levels, but hypercarbia is known to be the most important stimulus during tube breathing [8, 9]. We used the Mann–Whitney *U* test which is not universally accepted as post hoc test. There are several possible post hoc tests, but none is considered as gold standard.

In conclusion, we have demonstrated that infants whose mothers had second-hand smoke exposure during pregnancy had a similar damped ventilatory response to added dead space as infants whose mothers smoked antenatally.

## Data Availability

Data will be made available on reasonable request

## Abbreviations

CO_2_: Carbon dioxide
MMV: Maximum minute ventilation
MV: Minute ventilation
RR: Respiratory rate
SIDS: Sudden infant death syndrome
TC: Time constant
TV: Tidal volume

## Declarations

### Disclaimer

The views expressed are those of the authors and not necessarily those of the NHS, the NIHR or the Department of Health.
